# Recycled PET Composites Reinforced with Stainless Steel Lattice Structures Made by AM

**DOI:** 10.3390/polym15234591

**Published:** 2023-11-30

**Authors:** Mircea Rusu, Nicolae Balc, Marioara Moldovan, Stanca Cuc, Ioan Petean, Cosmin Cosma, Dan Leordean

**Affiliations:** 1 Department of Manufacturing Engineering, Faculty of Industrial Engineering, Robotics and Production Management, Technical University of Cluj-Napoca, 103-105 Muncii Blvd, 400641 Cluj-Napoca, Romania; mcrusu2206@yahoo.com (M.R.); nicolae.balc@tcm.utcluj.ro (N.B.); cosmin.cosma@tcm.utcluj.ro (C.C.); 2 Raluca Ripan Institute for Research in Chemistry, Babeș-Bolyai University, 30 Fantanele Street, 400294 Cluj-Napoca, Romania; marioara.moldovan@ubbcluj.ro (M.M.); stanca.boboia@ubbcluj.ro (S.C.); 3 Faculty of Chemistry and Chemical Engineering, Babes-Bolyai University, 11 Arany Janos Street, 400028 Cluj-Napoca, Romania; ioan.petean@ubbcluj.ro

**Keywords:** recycled PET, 316L stainless steel, additive manufacturing, metal reinforcement, IPC, interpenetrating phase composites

## Abstract

Polyethylene terephthalate (PET) recycling is one of the most important environmental issues, assuring a cleaner environment and reducing the carbon footprint of technological products, taking into account the quantities used year by year. The recycling possibilities depend on the quality of the collected material and on the targeted product. Current research aims to increase recycling quantities by putting together recycled PET in an innovative way as a filler for the additive manufactured metallic lattice structure. Starting from the structures mentioned above, a new range of composite materials was created: IPC (interpenetrating phase composites), materials with a complex architecture in which a solid phase, the reinforcement, is uniquely combined with the other phase, heated to the temperature of melting. The lattice structure was modeled by the intersection of two rings using Solid Works, which generates the lattice structure, which was further produced by an additive manufacturing technique from 316L stainless steel. The compressive strength shows low values for recycled PET, of about 26 MPa, while the stainless-steel lattice structure has about 47 MPa. Recycled PET molding into the lattice structure increases its compressive strength at 53 MPa. The Young’s moduli are influenced by the recycled PET reinforcement by an increase from about 1400 MPa for the bare lattice structure to about 1750 MPa for the reinforced structure. This sustains the idea that recycled PET improves the composite elastic behavior due to its superior Young’s modulus of about 1570 MPa, acting synergically with the stainless-steel lattice structure. The morphology was investigated with SEM microscopy, revealing the binding ability of recycled PET to the 316L surface, assuring a coherent composite. The failure was also investigated using SEM microscopy, revealing that the microstructural unevenness may act as a local tensor, which promotes the interfacial failure within local de-laminations that weakens the composite, which finally breaks.

## 1. Introduction

Plastic recycling stands as a pivotal solution in mitigating the relentless build-up of polymeric waste, thus curbing environmental degradation. The enormity of this industry, characterized by high volumes of low-weight polymers, once grappled with the challenge of effectively retrieving materials with substantial value, enough to offset retrieval costs. Bridging the divide between research-driven recycling solutions and their industrial implementation posed a significant hurdle. However, amid soaring prices of fossil-derived components, integral to polymer production, the spotlight has shifted towards pioneering and inventive recycling methodologies. This shift has been propelled by the urgency to circumvent environmental hazards linked to plastic waste and harness the potential economic benefits of sustainable practices. As sustainability gains traction, innovations in recycling are poised to revolutionize the industry, offering promising pathways towards a more eco-conscious and economically viable future.

Additive manufacturing gains a lot of advantages regarding the newest technologies due to its ability to generate complex structures from various materials such as polymers [1,2], ceramics [3,4] and metals [5,6], adding layer over layer during the printing process. This new technology is suitable for complex geometry [7,8] that would be very difficult for conventional casting or mechanical cutting [9].

Additive manufacturing has a lot of applications which depend on both used materials and the printing mode. Lattice structures are one of the newest approaches in additive manufacturing, and assume an elementary cell unit which is repeated through a graphic design model of the desired ensemble [10,11,12]. The elementary cell repeating mode would influence the mechanical properties of the printed structure. Thus, CAD models are very important for the lattice structure design and printer characteristics for structure manufacturing. A lot of lattice structures manufactured from biomaterials are used as scaffolds for tissue recovery [9,10].

Since 1940, polymer waste has been a significant issue that requires proper management to avoid environmental issues caused by low degradation under weathering conditions [13,14]. The technological waste resulting from the production flow should be automatically recirculated into the feedstock. The major problem arises along with used PET products and packages, which are more likely to become garbage instead of waste. Thus, their recycling implies proper sorting followed by adequate cleaning and conditioning [15,16]. The success of this management would generate proper feedstock for new PET products or for novel applications.

A lot of novel materials require both complex structures and robustness, which might be attained by combining special alloys processed by additive manufacturing with polymers. The metallic structure would bring increased mechanical strength, while polymeric reinforcement would enhance the elastic behavior [17,18]. Another advantage is achieved when the polymer can be substituted with its recycled version. Such beneficial property-embedding makes these materials suitable for vibration dampers or anti-earthquake protections [19,20]. This class of materials is known as interpenetrating phase composites (IPC) and there are at least two phases that are three dimensional interconnected, generating a topologically continuous network throughout the microstructure.

The novelty of the present research is the innovative use of recycled PET as filler for a 316L stainless steel lattice structure produced using a selective laser melting (SLM) technique. These samples are intended to be used for figuring out the mechanical behavior under compression strength and the subsequent Young’s Modulus. Another important aim of the current research is to establish the ability of recycled PET in assuring composite cohesion and mechanical strength.

## 2. Materials and Methods

### 2.1. Lattice Structures’ Design and Manufacturing

**Figure 1:**
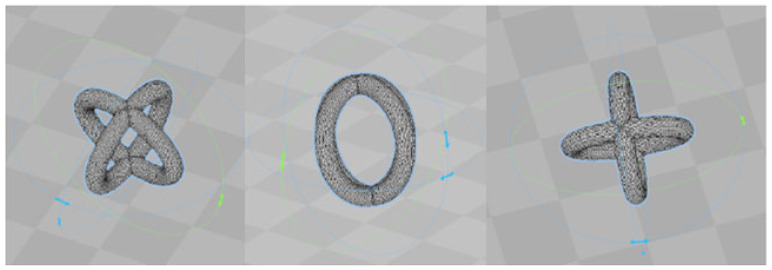
CAD model of the elementary cell.

The elementary cell was designed using Solid Works 2022 software produced by Dassault Systems, Vélizy-Villacoublay, France. It was generated by the perpendicular intersection of two identical rings, as shown in Figure 1. It is presented in different positions for better observation of the complex shape: left—isometric view, center—front view, and right—top view.

This structure is meant to be used without supports during SLM printing; in fact, each element is sustained by the previous one. The cross-section of the structural element is 0.3 mm and is circular in shape. The height of an elementary cell (double O-ring type) is 1.60 mm. The chosen dimensions are proper for the melted PET infiltration during molding. Thus, the elementary cell has a volume of 0.67 mm^3^ and a surface area of 8.75 mm^2^, according to the information provided by the Solid Works v2022 CAD software.

**Figure 2:**
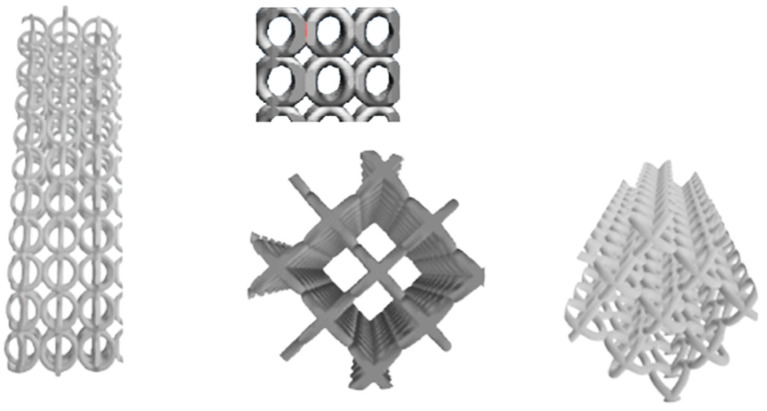
Cylinder designed using Magics software based on the elementary cell.

The process was repeated, forming a cylinder using Magics software for 3D printing produced by Materialise HQ, Leuven, Belgium. The cylinder diameter is 6 mm and its height is 18 mm (Figure 2). The elementary cells are connected to each other in all three directions. The degree of interpenetration is 0.1 mm.

The designed structure was printed with 316L stainless steel powder with spherical particles of about 50 µm diameter produced by MCP HEK Tooling, Lübeck, Germany. Thus, it will assure a theoretical porosity of 89%. This alloy is based on elements such as chromium, nickel, and molybdenum, which ensure a high stability. The complete chemical composition provided by the manufacturer is presented in Table 1.

**Figure 3:**
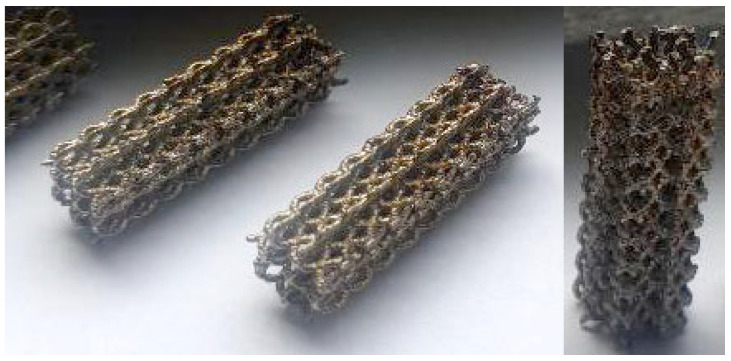
SLM-printed cylinder.

The modeled cylinder was printed through a SLM tridimensional printer ReaLizer 250 produced by ReaLizer GmbH, Borchen, Germany. The SLM printer was set up with the following parameters: laser power 180 W; scanning rate 1100 mm/s; powder bead thickness 35 μm. Figure 3 presents images of the manufactured cylinder.

The cord diameter is approximately 0.4 mm and was obtained using the above parameters for the infill and 120 W and 300 mm/s for the outer boundary, which causes a significant reduction in porosity. Thus, the theoretical porosity is about 89.17%, while the measured value is just about 84.20%. In consequence, the cylinder’s physical characteristics are a diameter of 6 mm and height of 18 mm according to the requirement for compression test samples, with a mean mass of 0.63 g.

### 2.2. Composite Samples’ Preparation

Recycled PET material was subjected to a serious selection process to remove labels and caps, as well as sorting according to the color. The viable material was ground twice until flakes of 0.2–10 mm were obtained with a relative density of 1.22 g/cm^3^. The resulting recycled PET powder was properly conditioned through washing procedures, including boiling in a sodium hydroxide solution to remove oil and fat traces, and then it was thoroughly cleaned and dried. The conditioned flakes were melted at 240 °C and mixed with 5.5% PEG as a viscosity moderator and 8% TiO_2_ powder as a microstructural filler.

**Figure 4:**
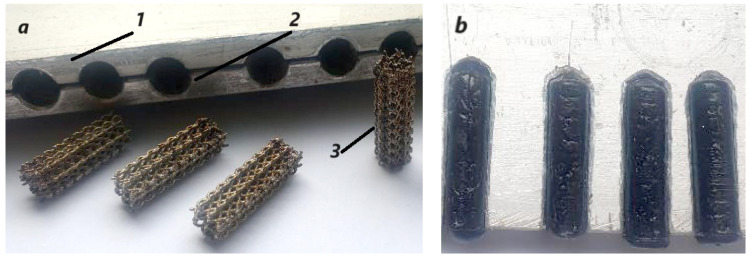
Composite samples’ preparation: (**a**) molding preparation and (**b**) obtained samples.

The composite samples were prepared using a two-sided mold, Figure 4a; sides are indicated with numerals 1 and 2. The mold sides were gathered and positioned into a vertical position and preheated at 100 °C, followed by the polymer injection into the samples slots at 240 °C. The SLM cylinders (3) were introduced afterwards into the mold slots while the mold was vibrated slightly to facilitate polymer penetration into the lattice structure pores. The samples were naturally cooled along with the mold until the ambient temperature was reached, when samples were extracted from the mold. Their aspect is presented in Figure 4b. Finally, we prepared the following samples for mechanical testing, as described in table. To begin with, samples S1 (Table 2) were taken into account, with recycled PET without any other addition, which we considered the standard. Samples S2 (Table 2) contained PEG and TiO_2_ to improve the adhesion between the recycled PET flakes and achieve denser structures compared to those of the S1 type. Samples S3 and S4 (Table 2) were made of lattice structures, the latter having impregnated inside its structure the composite material from S2 samples, which were superior to pure recycled PET from S1 samples.

### 2.3. Investigation Methods

The compression test was conducted according to the ASTM D790 and D638 standards using an Instron 8112 testing machine, Instron Company, Norwood, MA, USA. Five samples were tested for each composition and the mean value was further used in the current research. The Young’s Modulus was also calculated for the elastic behavior description.

Microstructural aspects were investigated using scanning electron microscopy, SEM, with an Inspect S50 SEM Microscope produced by FEI Company, Hillsboro, OR, USA. The samples were investigated in high vacuum mode and several magnifications were used: 100× for the general aspect, 500× for the rough microstructure, and 5000× for fine microstructural details.

Statistical analysis was effectuated with an Anova test followed by Tukey’s post hoc test with a significance level of 0.05; thus, *p* < 0.05 indicates significant statistical differences while *p* > 0.05 indicates no statistical differences.

## 3. Results and Discussion

**Figure 5:**
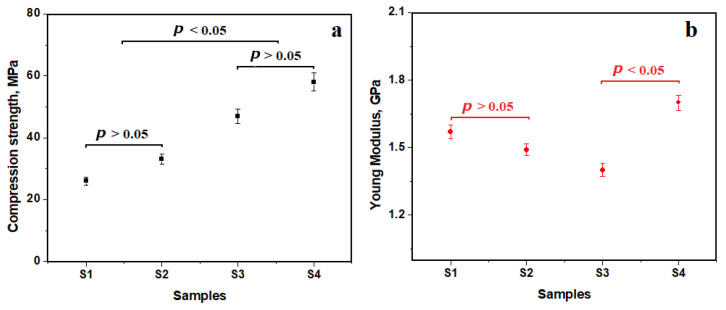
Results of the mechanical properties testing: (**a**) compression strength and (**b**) Young’s modulus.

Compression strength results show a value of about 26 MPa for the recycled PET samples, Figure 5a. The addition with PEG proved to increase the rheological behavior, while the embedding of nanostructural TiO_2_ powder caused a slight increase in compressive strength, up to 33 MPa. This fact is in accordance with data in the literature [21,22]. However, the Anova test followed by Tukey’s post hoc test showed a value greater than the significance level of 0.05, and thus there were no statistical differences.

An SLM cylinder made of 316L stainless steel (sample S3) has a compression strength of 47 MPa (Figure 5a). Similar aspects were reported in the literature [23,24]. The reinforced sample (S4) proved to be stronger than the bare SLM structure, resulting in a compression strength of 53 MPa. This reveals a synergic action between the SLM structure and recycled PET. The statistical analysis shows that both SLM and filled SLM structures belong to the same group without significant differences.

Significant differences occurred between the two observed groups: PET samples with low compression strength and samples with SLM structures with high resistance on the compression forces. The reinforcement of the selective laser melting (SLM) structure through recycled PET (rPET) might seem minimally impactful initially. However, looking more closely at the details shown in Figure 5b, an obvious trend emerges. Remarkably, the rPET-incorporated SLM structure showcases the highest recorded Young’s modulus, reaching approximately 1.7 GPa. This figure significantly exceeds the approximate value of 1.4 GPa shown by the unmodified SLM structure. The difference between these values carries statistical significance, a fact highlighted by the Anova test shown in Figure 5b.

This result highlights the immense potential of integrating recycled PET into SLM architectures, exhibiting not just a nominal improvement, but a substantial enhancement in structural integrity. Such findings support the deeper exploration of sustainable materials in advanced manufacturing techniques, signaling a promising direction for improving material properties without compromising ecological sensitivities. The observed leap in modulus, validated statistically, prompts a reevaluation of the efficacy and potential applications of rPET integration within SLM-manufactured parts.

It seems that rPET increases the elastic behavior of the composite structure besides the small increase in the compressive strength. The material with a high deformation modulus will take most of the load, while the recycled PET still plays an important role in deformation and crack evolution.

**Figure 6:**
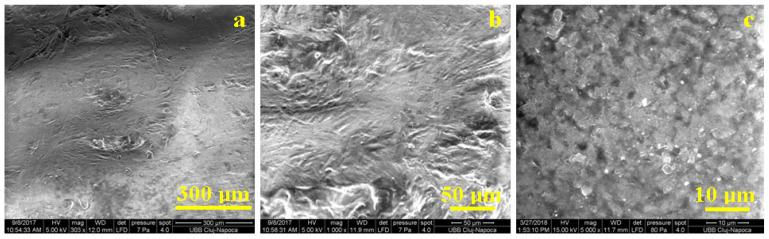
SEM images for S2 observed at different magnifications: (**a**) 300×, (**b**) 1000×, and (**c**) 5000×.

Microstructural aspects play a key role in the reinforcement success. Being a thermoplastic polymer, it can be heated and reshaped into an infinite number of shapes. With each reprocessing, the mechanical properties are slightly altered, which is why it is necessary to intervene. The addition of recycled PET is the first step. The low magnification investigation effectuated with an SEM microscope in Figure 6a revealed the overall aspect of the S2 sample, revealing a uniform and compact structure where the recycled PET embedded the filler particles uniformly and there was strong connectivity and bonding between the phases. Microscopic details in Figure 6b reveal the polymer flowing marks during the filler embedding, which indicates the efficiency of PEG acting as a viscosity moderator. The compactness of the microstructure is proved by the lack of superficial pores.

Figure 6c, observed at high magnification, reveals the fine microstructure of an S2 sample that contains clusters of nanostructured TiO_2_ with a rounded shape with irregular borders and a diameter ranging from 1 to 3 μm. These are very well embedded into the polymer matrix, indicating a very coherent material. The structure with the two phases acts as an iso-stress and iso-strain material.

**Figure 7:**
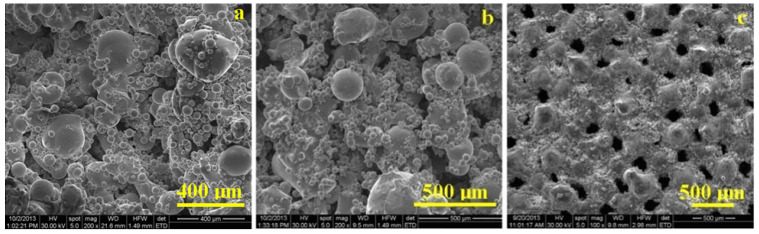
SEM images for the SLM cylinder reinforced with rPET containing nanostructural TiO_2_ filler: (**a**) unfilled SLM structure, (**b**) reinforced structure, and (**c**) superficial pores.

The SLM cylinder microstructure is also very important for the reinforcement success. Figure 7a evidences the microstructural details of the SLM elemental cell surface. The SLM procedure determines the stainless steel fine particles’ local melting and diffusion into bigger structures with rounded shapes and diameters ranging from 200 to 600 μm, interconnected by strongly developed diffusion necks. Their formation is facilitated by the heating regime [24]. On the surface of sintered structures appear attached un-melted stainless-steel particles with a spherical shape and sizes in agreement with the information from the producer. Such particles are also reported in the literature as being useful for facilitating the adhesion of several coatings [25,26].

The surface of the rPET reinforced SLM structure has a similar microstructure to the one observed for the un-filled one, but the details are less sharp due to the polymer layer penetration over the sintered stainless-steel structure (Figure 7b). The roughness induced by the presence of smaller particles attached to the diffused structures facilitates the polymer adhesion. Figure 7c reveals a particular area of the cylinder surface where several pores are visible. They were formed due to the solvent evaporation from the polymer during its solidification and the regular pattern is given by the pores within the SLM structure.

**Figure 8:**
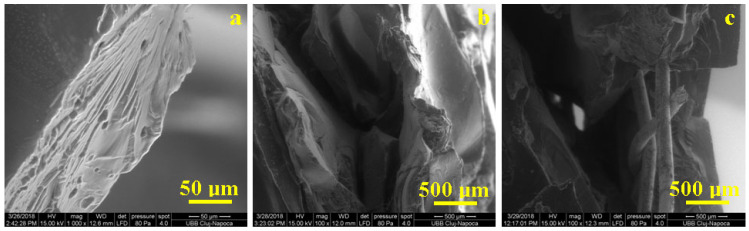
Fracture aspects for (**a**) S2, (**b**) rPET failure in S4, and (**c**) SLM structure failure in S4.

The compression of the S2 sample induces a strong deformation in the microstructure, and thus the polymeric chains are compressed vertically, which induces a lateral plastic deformation. Figure 8a reveals the smooth surface of the S2 sample in the left side of the image and the broken side on the center of the image. The force was applied vertically on the surface and the deformation lines were disposed horizontally, corresponding to the sample swelling. Several pores appeared inside the material, with a size of 5–15 μm and an elongated shape due to the sample deformation. These pores act as local tensors that initiate failure that propagates progressively on the deformation lines. This fact was also observed in the literature for recycled PET reinforced with metallic fillers [27,28].

The situation is more complex for S4 samples, where the polymeric filler tends to behave like an S2 sample, but the SLM structure disturbs the deformation mechanism. When the load increases progressively, the recycled PET is pressed onto the SLM metallic structure, which begins to section the polymer inside, acting as a failure promoter. The mechanism described is sustained by the microstructure in Figure 8b. The rPET broken parts presents harsh marks induced by the intensive friction with SLM structures during failure. The SLM structure is also strongly affected by the sample failure (Figure 8c); the circular elements within the structure become deformed wires without any resemblance to the initial shape and some rPET broken pieces are still attached to them.

The microstructural aspects evidenced by SEM microscopy are in accordance with the mechanical properties testing and show the efficacy of the recycled PET reinforcement of the SLM structures. The synergy between the recycled polymers and printed metallic structures facilitates a high compression strength along with a high elastic modulus, a fact in good agreement with the literature [29,30]. Increasing the Young’s Modulus during compressive solicitation makes these composite structures useful for various applications.

As an application, we propose the manufacturing of a bicycle pedal. Typically, these pedals are made from a solid metallic material with the addition of other polymeric materials. We have considered replacing the metallic parts with lightweight structures produced through SLM and substituting the polymeric parts with recycled PET. Using lighter parts for the moving ensembles fits the current trends due to energy saving strategies [31,32,33].

**Figure 9:**
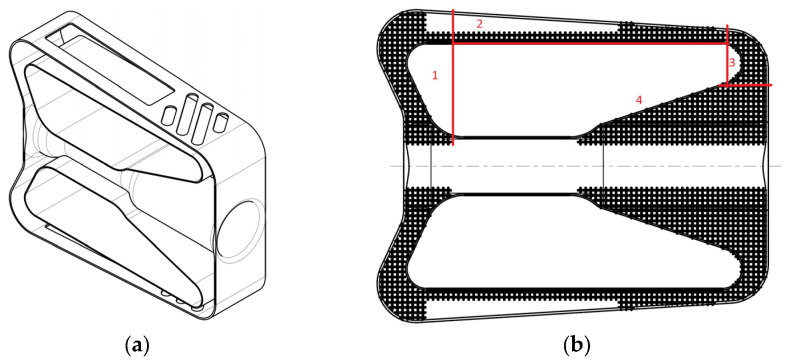

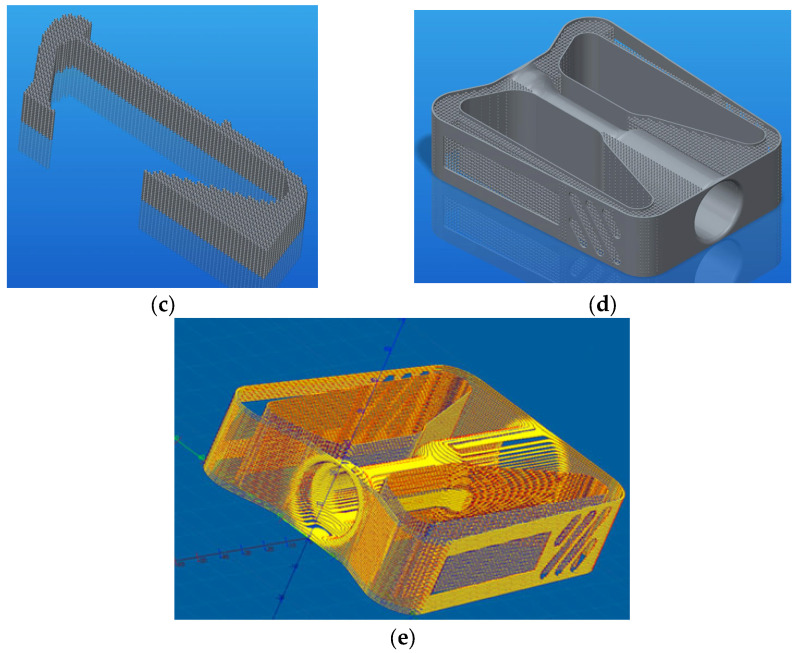
CAD model of a bike pedal manufactured from metallic SLM elements infiltrated with recycled PET: (**a**) bike pedal CAD model of structural framework, (**b**) bike pedal with all four lattice structures, (**c**) detailed view of one side of the lattice structure, (**d**) final CAD assembly and (**e**) computerized simulation for SLM manufacturing.

The first step was to model the attachment of the pedal elements and the geometric features that, together, form the structural framework (see Figure 9a). From this figure, it can be observed that there are two walls, one outer one measuring 0.6 mm and an inner one of 0.4 mm, between which there is a space that can be filled with rPET.

Creating a sturdy yet flexible structure for the recycled PET entailed a meticulous approach. The lattice design, an amalgamation of elementary cells outlined in Section 2, proved instrumental in fortifying the pedal’s integrity. The Creo Parametric v7 CAD software’s 3D Pattern function served as the crux for generating this lattice structure, a pivotal step toward reinforcing the pedal’s walls. Reference to Matus et al.’s work [34] underscores the significant impact of software calibration on printing precision, an aspect crucial to our endeavor. A fine-tuned pitch of 1.3 mm across all axes ensured an optimal overlap of 0.3 mm at cell intersections, fortifying the structure’s robustness.

However, computational limitations necessitated a pragmatic approach. Dividing the intricate lattice into four manageable elements became imperative, each crafted separately before unifying them into the final structure. This segmentation allowed us to navigate the computational constraints without compromising the intricacy and strength of the lattice design, as depicted in Figure 9c, showcasing one side of the pedal.

Completing the assembly was an exercise in mirroring the lattice structure, an ingenious method ensuring symmetry and coherence in the final pedal model (as demonstrated in Figure 9d). This meticulous approach not only bolstered the pedal’s durability, but also demonstrated the potential of recycled PET material in complex, functional designs. The integration of these methodologies offers insights into optimizing structures with recycled materials, paving the way for sustainable yet robust product development.

Upon the finalization of the pedal model, a comprehensive phase of computerized simulation ensued, primarily focusing on the manufacturing aspect through the utilization of SLM technology, as depicted in Figure 9e. This simulation phase aimed to intricately assess and optimize the production process, ensuring precision and efficacy in the utilization of SLM for fabricating the pedal.

In accordance with all the above statements, we consider that the purpose behind the design of this IPC, made of rPET and 316L stainless steel, respectively, to create a superior material that combines the most desirable properties of the two phases—high hardness and wear resistance of the metallic phase with the elasticity and elongation resistance of the polymer—was achieved. The infiltration of the melted polymer into the lattice structure without an external pressure led to the improvement of the properties of the structure in the three directions through the synergy between the two separate but totally interconnected phases. Redesigning the bicycle pedal and making it from these materials and through this technology is just an easy example of the vast possibilities for implementation.

Computerized simulation of the newly generated pedal indicates that it is feasible for manufacturing. This will be the next step for the current research in proving the efficacy of SLM structures reinforced with recycled PET for various industrial products.

## 4. Conclusions

The current research gives evidence for the benefits of SLM structures’ reinforcement with recycled PET by a small increase in the compressive strength and a significant increase in the elastic properties. The average values obtained for the compression strength of lattice structures reinforced with rPET in relation to those of 316L stainless steel were 13% higher, achieving an increase from 47 to 53 MPa. Also, with the same types of samples, we found the maximum value for Young’s modulus, increasing by 21% from 1.4 to 1.7GPa compared to those without reinforcement.

The values above confirm the superiority of the lattice structure with embedded rPET compared to the other samples and represent a starting point for numerous study possibilities in the field of recycled plastic materials. By redesigning some common, everyday elements, like the example above with the bicycle pedal, innovative solutions can be found to replace classic materials with an important component of care for the environment using a recycled polymer infiltrated into the internal structures of lattice structures. An important achievement of this structure is that it can be 3D multiplied and manufactured in many designs. Also, this innovative approach for polymeric recycled materials has the ability to go further with the applications in which these materials can be used. Thus, the resulting composite structure is more practical for various industrial applications that require good elastic properties of the components that should be lighter than common metallic parts. These aspects fit the current trends in the automotive industry [33,34,35].

Another important conclusion is that recycled PET conditioning prior to reinforcement is a very important step. Thus, the usage of PEG as a viscosity moderator and of the nanostructured TiO_2_ filler plays a key role in the success of the reinforcing process.

Each of the materials used, with their own distinct characteristics, contribute not only through intrinsic properties but also with a structure design that is responsible for the adhesion between the involved phases. This approach of infiltrating the metallic SLM structure with lower pressure using polymer can be used for the rapid fabrication of samples.

In the end, it is important to mention that the materials, architecture, and design parameters could provide a significant groundwork for research themes for recycling and materials science fields.

## Figures and Tables

**Table 1:** The 316L alloy chemical composition according to the manufacturer.

Chemical Element	C	Mn	Si	P	S	Cr	Mo	Ni	N	Fe
Wt.%	0.03	2	0.75	0.045	0.03	18	3	14	0.1	66

**Table 2:** Samples’ composition description.

Samples	Composition
S1	Recycled PET
S2	Recycled PET + 5.5% PEG + 8% TiO_2_
S3	SLM cylinder
S4	SLM cylinder filled with Recycled PET + 5.5% PEG + 8% TiO_2_

## Data Availability

Data are contained within the article.
